# Kif3a Guides Microtubular Dynamics, Migration and Lumen Formation of MDCK Cells

**DOI:** 10.1371/journal.pone.0062165

**Published:** 2013-05-01

**Authors:** Christopher Boehlke, Fruzsina Kotsis, Bjoern Buchholz, Christian Powelske, Kai-Uwe Eckardt, Gerd Walz, Roland Nitschke, E. Wolfgang Kuehn

**Affiliations:** 1 Department of Nephrology, University Hospital, Freiburg, Germany; 2 Department of Nephrology, University Hospital, Erlangen, Germany; 3 BIOSS Center for Biological Signaling Studies, Albert-Ludwig-University, Freiburg, Germany; 4 Life Imaging Center, Center for Biosystems Analysis, Albert-Ludwig-University, Freiburg, Germany; NCMLS, Radboud University Nijmegen Medical Center, Netherlands

## Abstract

The microtubular motor Kinesin-2 and its subunit Kif3a are essential for the formation of primary cilia, an organelle implicated in a wide spectrum of developmental abnormalities. Outside cilia, Kinesin-2 mediated transport has been implicated in vesicle and N-cadherin transport, but it is unknown if and how extraciliary Kif3a affects basic cellular functions such as migration or the formation of multicellular structures. Here we show that tetracycline inducible depletion of Kif3a in MDCK cells slows epithelial cell migration. Microtubules at the leading edge of Kif3a depleted cells failed to grow perpendicularly into the leading edge and microtubular dynamics were dampened in Kif3a depleted cells. Loss of Kif3a retarded lateral membrane specification and completely prevented the formation of three-dimensional spheres in collagen. These data uncover that Kif3a regulates the microtubular cytoskeleton in the cell periphery and imply that extra-ciliary Kif3a has an unexpected function in morphogenesis.

## Introduction

Kif3a is a subunit of hetero-trimeric Kinesin-2, a microtubule (MT) plus-end directed motor protein [1]. Hetero-trimeric Kinesin-2 consists of the two motor subunits Kif3a/Kif3b and the cargo-binding protein Kap3 [2]. Kinesin-2 has been mostly studied in cilia where it functions to carry protein complexes along the microtubular axoneme from the base to the tip of the cilium in a process termed intraflagellar transport (IFT) that is required for cilia formation [3]. The constitutive deletion of Kif3a in mice results in severely disordered mesoderm development reminiscent of defective hedgehog signalling and in mid-embryonic lethality [4], [5]. Several Hedgehog (HH) components localize to cilia and this organelle plays a central role in the activation of the HH pathway which could explain the similarities in HH and cilia phenotypes [5], [6]. Yet, several lines of evidence suggest that kinesin-2 mediated transport occurs outside cilia: Expression of mutant Kif3a in cultured Xenopus cells blocked vesicle transport from the ER to the Golgi preventing pigment dispersion, and deletion of the kinesin-2 component Kap3 was associated with decreased transport of N-cadherin to the cell periphery [7], [8]. Interestingly, the homodimeric kinesin-2 subunit Kif17, which cooperates with Kif3a in ciliary transport of *C. elegans* sensory neurons, affects the polymerization and stability of microtubules and has been implicated in epithelial morphogenesis [9]. These observations raise the possibility that Kif3a and hetero-trimeric kinesin-2 play a role in microtubular transport and MT dynamics also outside the cilium.

Microtubular dynamics are an essential component of cell migration, which requires the perpetual reorganization of the microtubule network in a dynamic process that involves capture of MT plus-ends at the cortex and the generation of pulling forces by the minus end directed motor dynein [10]. MT capturing is orchestrated by several plus-end binding factors such as proteins of the Eb (end binding), Clip (CAP-GLY domain-containing linker protein), and Clasp (Clip associated protein) families and through APC (adenomatous polyposis coli protein) [11]. These TIP-proteins orchestrate microtubule dynamic instability, which refers to a process where MT plus-ends undergo cycles of polymerisation and depolymerisation resulting in MT-growth, shrinkage or pausing [12]. Only sparse information exists on the role of microtubules and dynamic instability in epithelial morphogenesis. Depletion of the TIP-protein Eb1 by shRNA disrupted MT organization and interfered with epithelial remodelling in a 3D matrix [13]. However lumen formation in matrigel was only moderately affected, suggesting a complex relationship of MT dynamics and the formation of polarized three-dimensional structures that needs to be explored.

We report here that depletion of Kif3a slows the migration of MDCK cells. Kif3a depleted cells have smaller leading edges. The directionality of MTs into the leading edge is disturbed and MT dynamics are severely altered. Kif3a contributes to lateral membrane specification and is required for lumen formation in collagen. Our findings demonstrate that Kif3a has unexpected functions in MT behaviour that are important for cell migration and epithelial morphogenesis. We suggest that MT dynamics may play an important role in epithelial lumen formation.

## Materials and Methods

### Cell culture and transgenic cell lines

MDCK cells were grown at 37°C, 21% O_2_ and 5% CO_2_, and maintained in Dulbecco's modified Eagle's medium (DMEM), 10% FBS, and 1% penicillin/streptomycin. For cells stably expressing non-inducible fusion proteins 2.5 mg/ml geneticin was added. A lentiviral system was used for the inducible knowdown of Kif3a [14]. MDCK cells were first transduced with lentivirus encoding the tetracycline sensitive tTR-KRAB repressor and a dsRed reporter [15]. A second transduction step followed with lentivirus encoding a shRNA against Kif3a (Kif3a-i1: 5′-AGGCTAGAGCTGAATTAGAG-3′) and (Kif3a-i2: 5′-GCAAGAACGCTTGGATATT-3′) plus a GFP reporter (pLVTH vector), both under the control of tTR-KRAB. Control cells were obtained in a similar process, except that the lentivirus for the second transduction was prepared with pLVTH without shRNA or pLVTH with shRNA against luciferase (Luci-i: 5′-CGTACGCGGAATACTTCGA-3′). The Kif3a-rescue cell line was obtained as follows: using Mlu and Not restriction sites, human Kif3a was cloned into pLVTH which also contained Kif3a-i1. The target sequence of shRNA 1 was altered at three sites by site directed mutagenesis (5′-GAAACAAAGCTAGAGCTGAGCTAGAGAAACGGGAAAAAG-3′) maintaining the amino acid sequence. For the MDCK.Kif3a-i1/EB1-YFP cell line Eb1-YFP (pLXSN) [16] was retrovirally transduced into MDCK.Kif3a-i1. For the MDCK.Kif3a-i/KIM1-CFP cell line KIM1-CFP was cloned into pLXSN and used for retroviral transduction of Kif3a-i1 MDCK cells [16]. For the MDCK.Kif3a-i/α-Tubulin-YFP cell line human α-Tubulin was cloned (Mlu, Not) into pLXSN with a C-Terminal YFP and again used for retroviral transduction of Kif3a-i1 MDCK cells.

### Western blot

The following antibodies were used: anti-GFP (Santa Cruz, SC9996), anti-gamma-Tubulin (Sigma-Aldrich, T6557), anti-Flag (Sigma-Aldrich, M2), anti-Actin (Sigma-Aldrich, A1978), anti-acetylated-Tubulin (Sigma-Aldrich, T6793), anti-Glu(detyrosinated)-Tubulin (Millipore, AB3201), anti-Kif3a (BD Transduction Labs, 611508) and anti-Kap3 (Santa Cruz, SC-135958). Cells were grown in 10 cm cell culture dishes. Cell lysis and Western blot were performed as described [14]. Prior to blotting, the membranes were fixed in glutaraldehyde.

### Ca^++^ switch and TEER measurements

MDCK cells were plated with a density of 7.5×10^4^ cells per square centimeter on transwell filters with a pore size of 0.4 µm (Corning Costar, Corning, USA). After 5 days of incubation in the presence (+Tet) and absence of tetracycline (-Tet), respectively, Kif3a-i cells and pLVTH (empty vector) cells were incubated in Minimal Eagle Essential Medium for 16 hours. After switching back to Dulbecco's modified Eagle's medium (DMEM), 10% FBS, and 1% penicillin/streptomycin, transepithelial electrical resistance (TEER) was measured using an epithelial volt-ohmmeter at indicated time points (Endohm-12/EVOM, World Precision Instruments, Sarasota, FL).

### Wound healing experiments

For wound healing assays, MDCK cells were plated and grown to confluence on Ibidi µ-dishes (Ibidi, Munich, Germany) coated with collagen A (Biochrom AG, Berlin, Germany). For quantification of migration speed, cells were incubated for 2 days±5 µg/ml tetracycline and wounded using a micropipette. Cells were imaged using a Nikon Biostation IM (Nikon, Melville, USA), which includes a CO_2_ incubation chamber equipped with a heating unit and a motorized xy-table. Migration speed was calculated using the automatic tracking software of NIS ElementsSystem (Nikon, Melville, USA). Imaging of Kif3a-i/EB-1–YFP and Kif3a-i/α-Tubulin-YFP cells for microtubule tracking was performed in temperature controlled conditions under an inverted microscope (Axiovert 200 Microscope with objective Plan-Apochromat 100×/1.4Oil, Carl Zeiss MicroImaging, Germany).

### Cystogenesis and tubulogenesis of MDCK cells

Kif3a-i cells and pLVTH (empty vector controls)- cells were trypsinized and resuspended as a single-cell suspension of 1–2×10^4^ cells/ml in type I collagen solution (PureCol, Leimuiden, Netherland) [17]. 400 µl of this collagen cell suspension were placed in each well of a 24-well plate receiving 4000–8000 single cells/well for experiments including HGF treatment or onto ibidi “ibitreat” µ-slides (iBiTreat, ibidi GmbH, Munich, Germany) to quantify cyst formation. Tubulogenesis was initiated after 2–3 days of *in vitro* cystogenesis by administration of 10 ng/ml Hepatocyte Growth Factor (HGF, ImmunoTools, Friesoythe, Germany) to the culture media for 24 h. Tubule-like extensions were counted from 20 randomly chosen cysts at 100×magnification at the centre of each well. 6–9 wells per condition were examined. 1 ml of culture medium ±5 µg/ml tetracycline was added to each well and changed every 48 h, and analyzed with a Leica DMR microscope (Leica Microsystems, Wetzlar, Germany) using differential interference contrast. Photographs were digitally recorded by means of a Nikon Digital Sight DS-U1 system. Quantifications of spherical structures with ibidi µ-slides involved 150 randomly chosen cysts from three independent experiments at day 6–7. Only structures defined by a single lumen were counted.

### Confocal microscopy

2D-Cultures: MDCK.Kif3a-i cells and Kif3a-i/KIM1-CFP cells were grown on glass coverslips for 4 days in the absence or presence of tetracycline. Kif3a-deficient cells were fixed and immunofluorescence staining was performed as described later. 3D-cultures: Kif3a-i/KIM1-CFP cells were grown in collagen I for 7 days in the absence or presence of tetracycline, fixed, stained and imaged. Confocal microscopy was performed using a LSM 5 Life Duo equipped with C-Apochromat 40×/1.2NA and LCA Plan-Neofluar 63×/1.3NA water objectives and an α-Plan-Neofluar 100×/1.45NA oil objective (all Carl Zeiss MicroImaging GmbH, Jena, Germany) with the pinhole set to 1 µm optical slice thickness. Excitation of the fluorophores (Hoechst 33342, Cerulean, Alexa-488, eGFP, DsRed2, Cy5) was performed at 405, 458, 488, 561, and 633 nm respectively. For detection of the emission signal at specified ranges, the spectral meta detector or normal photomultiplier channels were used with BP filter 420–480, 463–485 (meta detector), BP 505–530, BP 575–615, LP 650 nm. Image analysis and 3D reconstruction were performed using Imaris Software (Bitplane, Zurich, Switzerland). For the z-cross section of Kif3a-i/KIM-CFP cells plated on solid support, 17 planes with an optical slice thickness of 0.5 µm, z-distance 0.5 µm, and a pixel time of 1.6 µs were acquired. All other confocal images were acquired with 1.0 µm optical slice thickness.

### Immunofluorescence

2D/3D cultures stainings were done using 4% paraformaldehyde or methanol/acetone (1∶1) depending on the antibody. Cells were permeabilized with 0.1% Triton X-100 in PBS and incubated in blocking solution (5% horse serum or 0.2% gold fish gelatine). Primary antibodies: mouse anti-acetylated-Tubulin (Sigma-Aldrich, T6793, 1∶3000), mouse anti-α-Tubulin (Sigma-Aldrich, T6199, 1∶200), rabbit anti-Glu(detyrosinated)-Tubulin (Millipore, AB3201; 1∶200), rabbit anti-Par3 (Millipore, 07–330, 1∶200), rabbit anti-APC (mAPC kindly provided by Inke Naethke, 1∶1000); mouse anti-APC (Abcam, AB58, 1∶200), mouse anti-E-cadherin (BD Transduction Lab., 610181; 1∶200), β-catenin (BD Transduction Lab., 610154, 1∶200), rabbit anti-ZO1 (Invitrogen, 339100, 1∶1000), Phalloidin488 (Santa Cruz, SC-363791, 1∶200) and Hoechst 33342. Antibodies were visualized using Cy5, Cy3-or Alexa-488-labelled secondary antibodies at a dilution of 1∶1000 (Jackson Immunoresearch).

### Eb1-tracking

Eb1 comets in Eb1-YFP expressing MDCK.Kif3a-i cells were imaged as described before under an inverted microscope (4D) and deconvolved using Huygens Essential software (Version 4.1, Scientific Volume Imaging, Hilversum Netherlands). The Eb1 trails were visualized in Imaris software (version 5.7.2, Bitplane, Zurich, Switzerland) by projecting consecutive time points and track length measurements were performed manually. Statistics were calculated with Excel (Microsoft).

### TIRF and measurements of microtubule dynamics

TIRF (total internal reflection microscopy) was performed with a Laser TIRF 3 system on an Axio-Observer Microscope (Carl Zeiss MicroImaging GmbH, Göttingen, Germany). MDCK.Ki3a-i/α-Tubulin-YFP and MDCK.Kif3a-YFP/α-Tubulin-CFP cells were plated into µ-dishes with a glass bottom (ibidi GmbH, Munich, Germany) incubated for two days in ± tetracycline conditions, scratched, and mounted on an incubation stage (TokaiHit, Shizuoka-ken, Japan) with 5% CO_2_ incubation and stable temperature at 37°C. Representative leading edge cells of each condition were imaged 6 hours after wounding at 5–10 s intervals using the 453/514 nm lasers. Microtubule dynamic instability parameters were calculated with Excel (Microsoft Corporation).

### Data analysis and statistics

Statistical analysis was carried out in EXCEL (Microsoft Cooperation). Statistical significance was calculated by unpaired t-test where not further indicated. All values are given as mean ± s.e.m. (standard error of the mean). P values of less than 0.05 were considered to be statistically significant.

## Results

### Kif3a mediates cell migration

**Figure 1:**
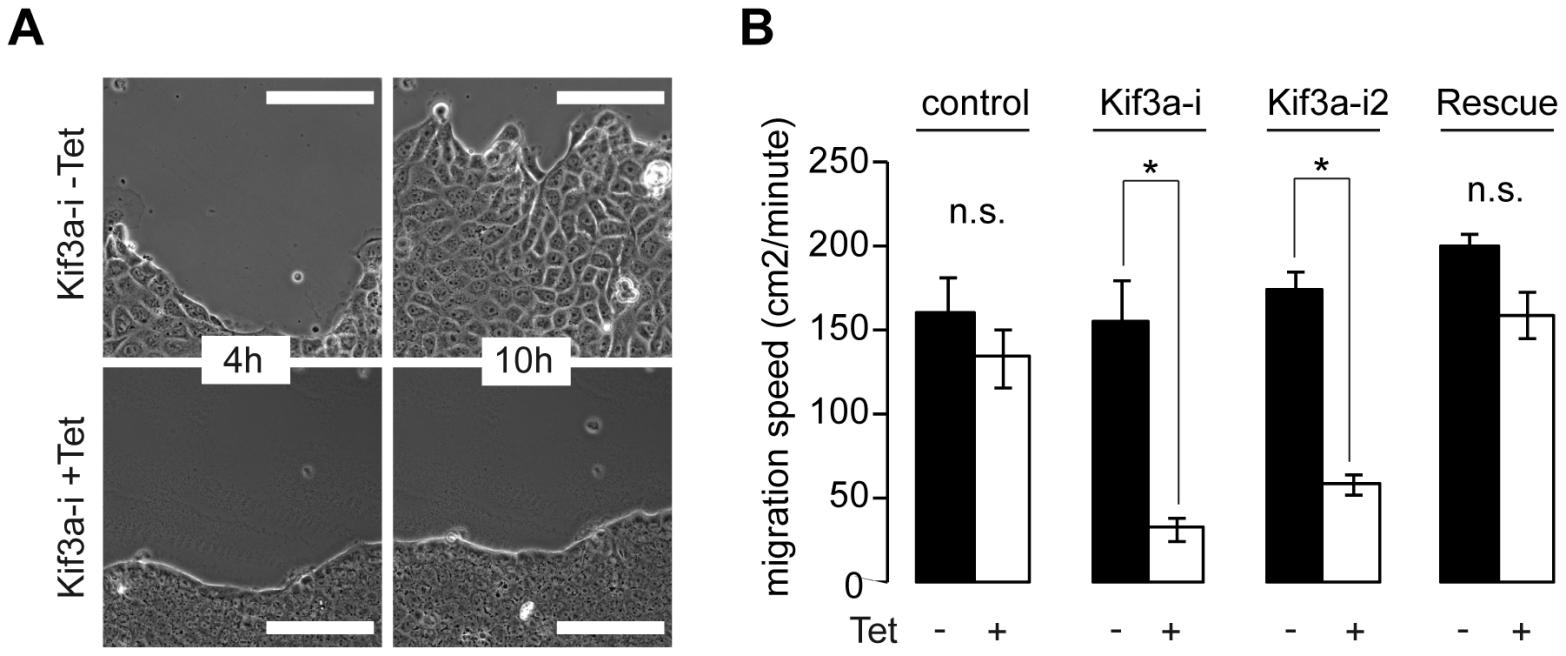
Depletion of Kif3a affects sheet migration. (**A**) Kif3a-i cells after 48 h with or without Tet were wounded and migration was observed over time (Video S1). Representative phase contrast images after 4 and 10 h. The Kif3a depleted cells appear denser and smaller. Scale Bars: 100 µm. (**B**) Quantification of migration speed in the presence or absence of Tet of control cells (Luci-i), 2 different Kif3a-i cell lines and rescue cells (details in text). The area (cm^2^) filled in during migration was measured semi-automatically (cm^2^/minute). − and+Tet: controls: 160±22% vs. 134±17%; Kif3a-i1: 155±25% vs. 32±7%; Kif3a-i2: 174±11% vs. 58±6%, Rescue: 200±9% vs. 160±14%, n = 3 each (>5 fields of view per n), * P<0.01, “n.s.” not significant.

To investigate Kif3a function in cells, we used lentivirally transduced MDCK cells that allow tetracycline inducible expression of a shRNA specific for Kif3a (Kif3a-i) [18]. After incubating these cells for 48 h with tetracycline, Kif3a protein was effectively depleted (Figure S1A). We assessed collective cell migration in Kif3a-deficient MDCK cells after the wounding of confluent monolayers 48 h after plating (Figure 1A, Video S1). Kif3a-deficient cells migrated markedly slower than cells in the absence of tetracycline, whereas tetracycline-treatment of control cells expressing shRNA against luciferase (Luci-i) [18] only had a marginal effect on wound closure (Figure 1B). To exclude off-target effects of the shRNA against Kif3a we created a second cell line using a different inducible shRNA with a separate target sequence (Kif3a-i2) (Figure S1B). Analysis of this cell line in wound healing assays again showed slower migration (Figure 1B). In a different approach, we expressed mutated, non-degradable human Kif3a in the Kif3a-i1 cells (Figure S1C) and found that the migration defect was rescued (Figure 1B). These findings demonstrate that Kif3a is required for effective cell migration.

### Kif3a associates with MTs at the leading edge

**Figure 2:**
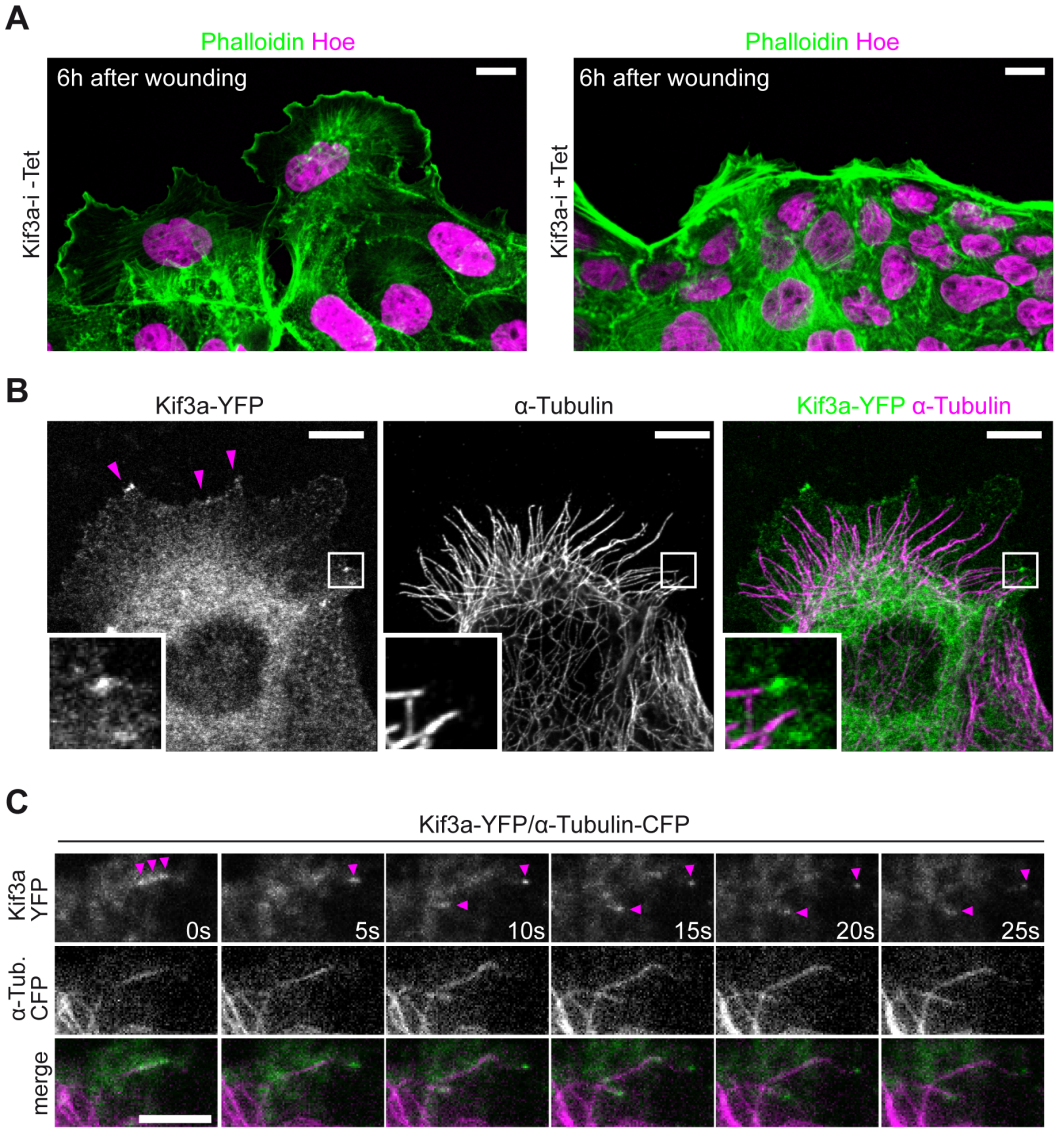
Kif3a associates with MTs at the leading edge. (**A**) MDCK.Kif3a-i cells were grown to confluency (48 hours), fixed 6 hours after wounding and stained for actin (phalloidin, green) and the nucleus (Hoechst, magenta). Cell protrusions appear markedly smaller in +Tet conditions. Scale Bars: 10 µm. (**B**) Migrating MDCK cells expressing Kif3a-YFP (green) were fixed and stained for α-tubulin (magenta). Kif3a-YFP localizes at microtubule tips and cell protrusions (magenta arrows). Scale Bars: 10 µm. (**C**) MDCK cells stably expressing Kif3a-YFP (green) and α-Tubulin-CFP (magenta) were imaged by dual color TIRF microscopy (Video S2). Kif3a-YFP dynamically co-localizes with growing MTs. Magenta arrows point to Kif3a-YFP accumulations along MTs and MT-tips. Scale Bars: 2 µm.

Kif3a depleted cells appeared strikingly denser and less spread (Figure 1A). On higher magnification much fewer lamellipodia were observed at the migrating front of Kif3a depleted compared to Kif3a expressing control cells (Figure 2A). We wondered if Kinesin-2 can be detected in lamellipodia. As has been described by others we were unable to detect endogenous Kif3a with existing antibodies [9]. Instead we investigated the localization of Kif3a-YFP and found patches of Kif3a at MT tips and membrane protrusions in fixed Kif3a-YFP expressing cells stained with an antibody against α-Tubulin (Figure 2B). To exclude staining artefacts, we utilized live cell imaging and examined microtubules and Kif3a movements by dual colour TIRF microscopy (Figure 2C, Video S2). We transfected MDCK cells with Kif3a-YFP and α-tubulin-CFP and observed Kif3a signals moving at the tips of a subset of growing MTs. These findings establish that Kif3a associates with dynamic MT in lamellipodia and prompted us to search for a function in MT behaviour.

### Depletion of Kif3a results in a paucity of MTs at the leading edge, altered MT orientation and decreased MT dynamicity

**Figure 3:**
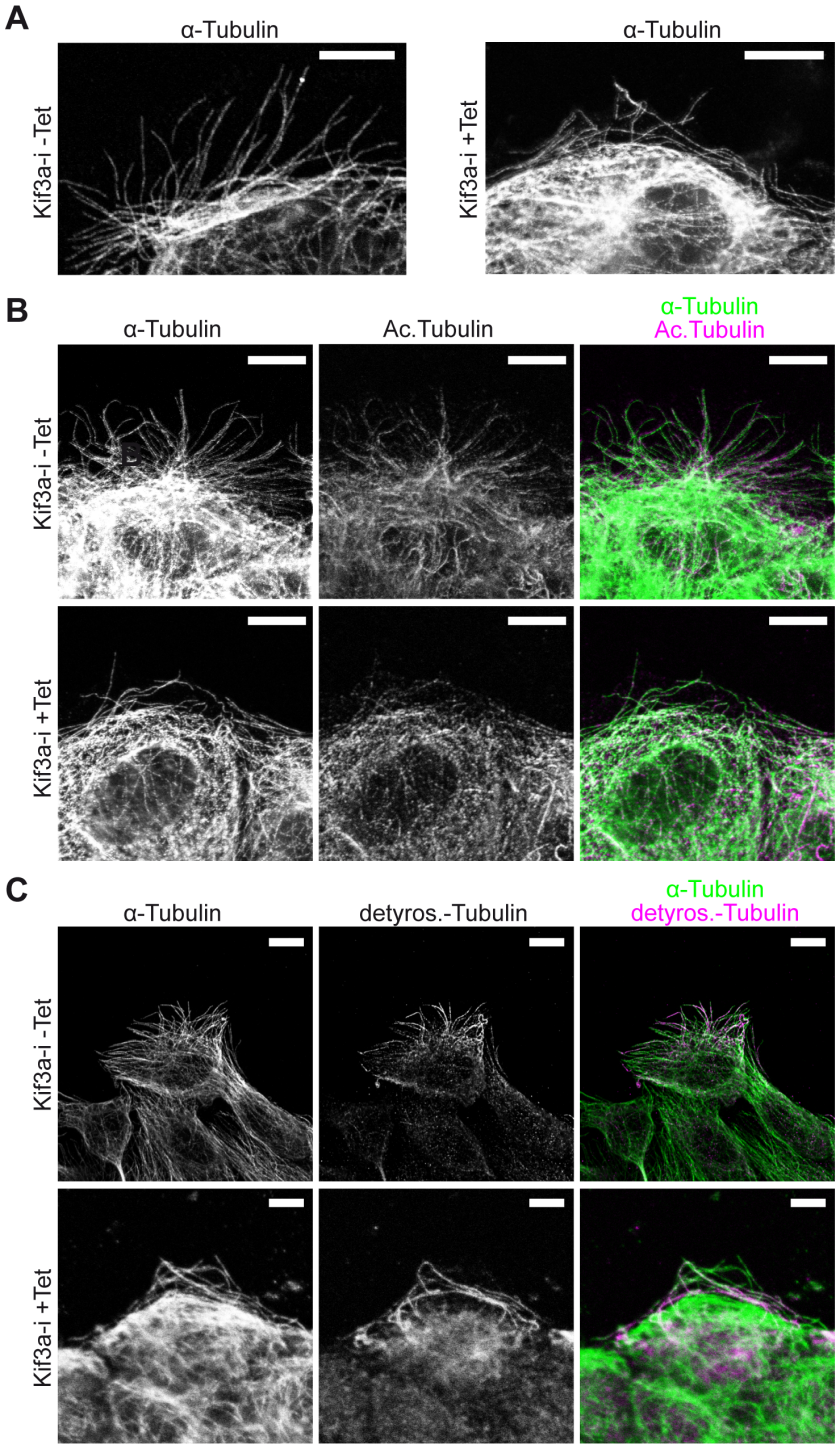
Kif3a directs microtubules, but does not affect MT post-translational modifications. (**A**) MDCK.Kif3a-i cells were grown to confluency (48 hours), fixed 6 hours after wounding and stained α-tubulin (white). MT directionality appears perturbed by Kif3a depletion (+Tet). Scale Bars: 10 µm. (**B**) Co-staining of α-tubulin (green) and acetylated tubulin (magenta) in MDCK cells shows no difference in acetylated microtubules in control cells (−Tet) compared to Kif3a-deficient cells (+Tet). Scale Bars: 10 µm. (**C**) Co-staining of α-tubulin (green) and detyrosinated tubulin (magenta) in MDCK cells shows no difference of detyrosinated microtubules in control cells (−Tet) compared to Kif3a-deficient cells (+Tet). Scale Bars: 10 µm.

**Figure 4:**
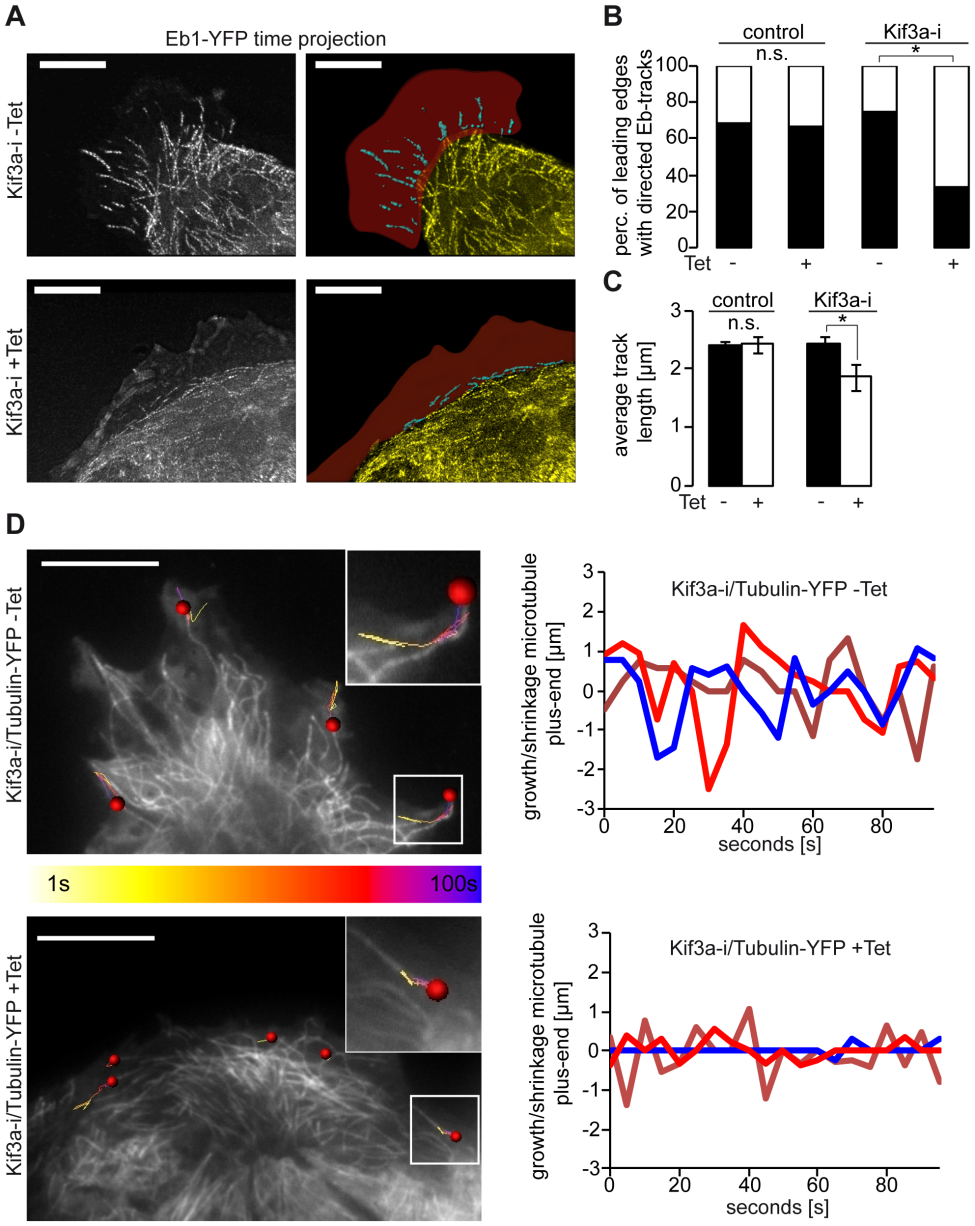
Kif3a affects MT dynamicity and directionality. (**A**) Eb1 comets in Eb1-YFP expressing MDCK.Kif3a-i cells (Video S3) were imaged and the Eb1 trails visualized by projecting consecutive time points. Lamellipodia are shown in red. Eb1 comets track away from the cell into the protrusion in the absence of tetracycline. In contrast, Eb1 tracks align in parallel to the cell body in Kif3a depleted cells (+Tet). Scale Bars: 10 µm. (**B**) The number of leading edges with directed Eb1-Tracks is unaffected by tetracycline in control cells (Luci-i) but is decreased when Kif3a is lost (Kif3a-i+Tet). − and + Tet: Kif3a-i: 79% vs. 27% and control: 71% vs. 65%; Chi-Square-Test *P<0.01. “n.s.” not significant. (**C**) The average length of Eb1-tracks was unchanged in control cells with tetracycline (Luci-i), but was decreased when Kif3a was depleted (Kif3a-i +Tet). − and+Tet: Kif3a-i: 2.4±0.1 µm vs. 1.9±0.2 µm, control: 2.4±0.1 µm vs. 2.4±0.1 µm *P<0.01. “n.s.” not significant. (**D**) TIRF microscopy of Tubulin-YFP in MDCK.Kif3a-i cells (Video S4). Images: Color coded trajectories of MT tips (yellow colour indicates the beginning of the track, purple colour the end). The trajectories are shorter in Kif3a depleted cells (+Tet, bottom). Graphs: Dynamic instability of representative MT tips is plotted over time. In Kif3a-deficient cells (+Tet, bottom) growth and shrinkage are markedly diminished compared to control (−Tet). For quantitative analysis see Table S1. Scale bars: 10 µm.

Staining against α-tubulin in Kif3a-deficient cells revealed a prominent finding: Instead of MTs leading straight into lamellipodia, the MTs in Kif3a depleted cells were mostly diverted at low angles laterally to the cell protrusions and the protrusions covered a smaller area (Figure 3A–C). On the other hand, MT stability, as assessed by MT tyrosination or glutamylation (Figure 3B, C and S1D) or nocodazole resistance (data not shown) were unaltered, although no APC clusters were detected at MT tips of Kif3a depleted cells (Figure S1E). To further analyze the differences in MT patterns at the leading edges of Kif3a depleted cells, we monitored MT growth by video microscopy of Eb1-YFP in Kif3a-i cells. This revealed a normal pattern of Eb1 at MT tips (Video S3). However, the Eb1 tracks failed to lead into the protrusions and instead moved laterally to the leading margin of the migrating cells (Video S3, Figure 4A and B). In addition, the Eb1-tracks were markedly shorter in Kif3a-deficient compared to control cells (Figure 4C), which suggested that the MTs were growing more slowly when Kif3a was depleted.

During cell migration MT plus-ends undergo cycles of polymerisation and depolymerisation which is referred to as dynamic instability [12]. This process is characterized by MT-growth, shrinkage and pausing. Our findings of slower Eb1 tracking in Kif3a depleted cells suggested that Kif3a plays a role in the MT growth rate. We therefore investigated MT behaviour in live cells by TIRF microscopy and found pronounced alterations in microtubule dynamics in Kif3a-deficient cells (Figure 4D, Video S4). Correlating with shorter EB1 tracks, the MTs of Kif3a depleted cells had a lower growth rate (Table S1). Intriguingly, also the shortening rate was reduced. In addition, the overall time spent growing and shrinking was strongly reduced in Kif3a depleted cells, resulting in an increased amount of time spent pausing (Figure 4D and Table S1). These findings demonstrate that Kif3a profoundly affects MT plus end dynamics, which may translate into orchestrating MT architecture at the leading edge of migrating cells.

### Kif3a-deficiency compromises the dynamics of tight junction assembly

**Figure 5:**
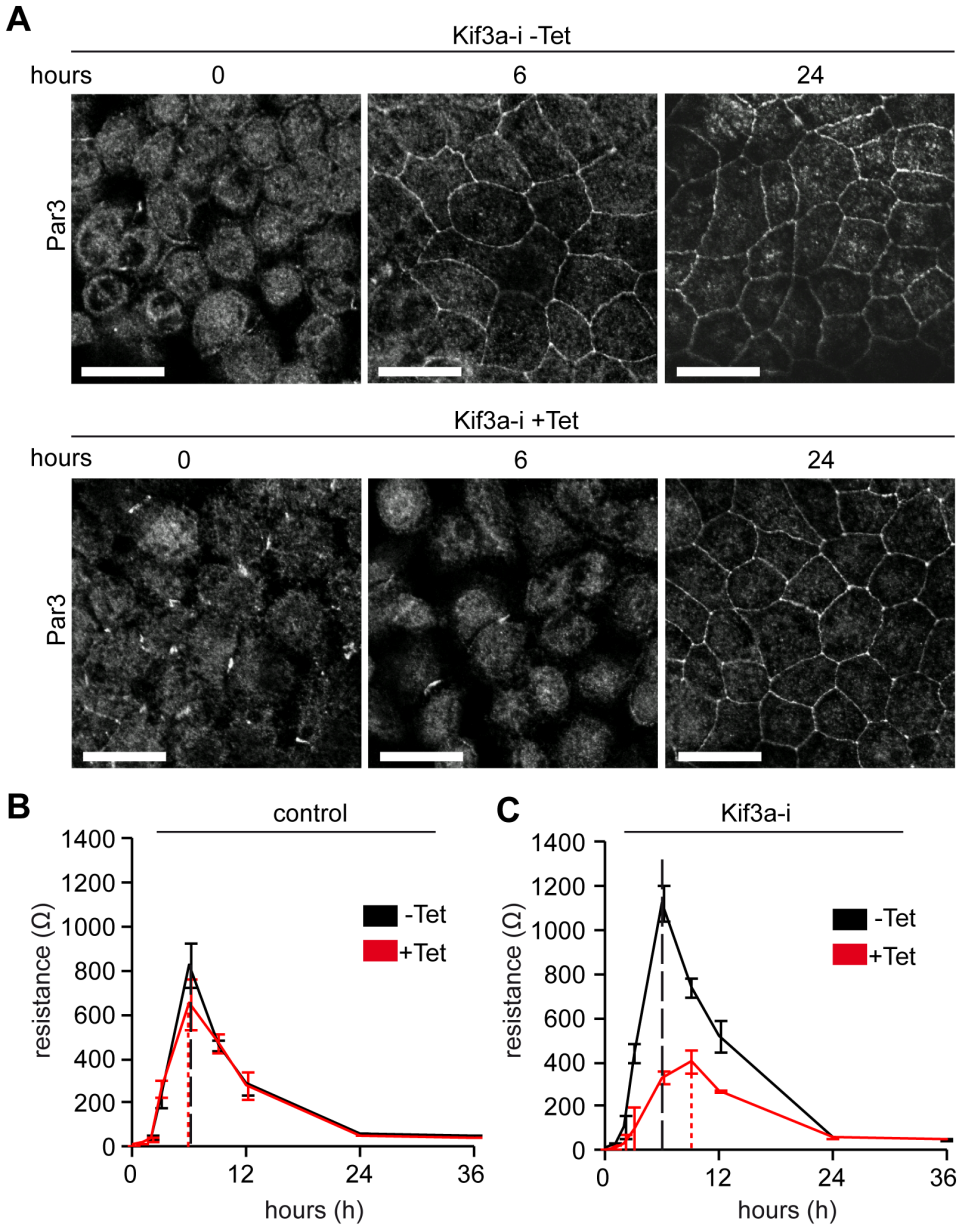
Tight junction assembly depends on Kif3a. (**A**) Confluent MDCK cells were subjected to calcium switch and stained for Par3 at the indicated time points. Kif3a depleted cells (+Tet, lower panels) reveal delayed appearance of Par3 at the lateral membrane compared to cells in the absence of tetracycline (−Tet, top panels). Scale Bars: 20 µm. (**B**) Transepithelial Electrical Resistance (TEER) measurement after Ca-switch to assess tight junction assembly. Control cells (pLVTH) upon tetracycline treatment (red curve, +Tet) show maximal resistance at the same time point compared to −Tet conditions (black curve). n = 6. (**C**) The peak of TEER after Ca-switch is delayed (9 h vs. 6 h) in Kif3a-i cells treated with tetracycline (+Tet, red line) versus controls (−Tet black line). n = 6.

Despite the strong migratory phenotype, apico-basal polarity seemed unperturbed in monolayers of Kif3a-deficient cells (Figure S2). Nonetheless, MTs are required for tight junction integrity [19], [20] so we wondered if Kif3a plays a role in the formation of the lateral membrane. The removal of calcium from the medium of cultured epithelial cells causes a breakdown of the tight and adherent junctions that is reversible after restoring calcium to the medium and involves Par3 [21]. When we depleted calcium in non-induced Kif3a-i MDCK cells, Par3 disappeared from the lateral membrane and re-appeared 6 hours after the re-addition of calcium (−Tet, Figure 5A). This was accompanied by a transient increase in transepithelial resistance (TEER, Figure 5B) [22]. In Kif3a depleted cells (+Tet), however, re-establishment of Par3 at tight junctions was delayed, as was the peak increase of TEER (Figure 5A and C), whereas the TEER peak was unaltered in tetracycline treated control cells. These findings suggest that the role of Kif3a on MTs during migration extends to the formation of epithelial monolayers, but that the defects in cell polarization caused by Kif3a depletion can be overcome in two-dimensional culture.

### Kif3a-deficient MDCK cells fail to form lumina in collagen

**Figure 6:**
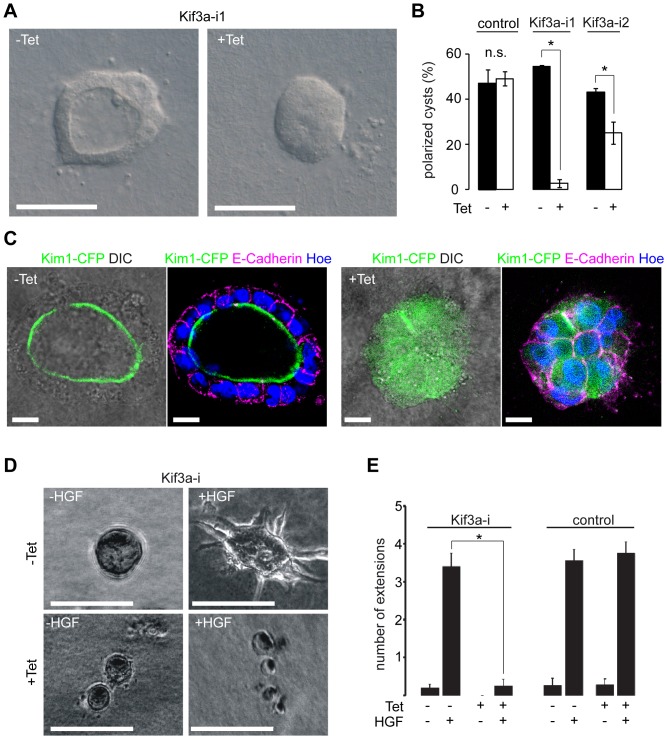
Lumen formation in epithelial morphogenesis requires Kif3a. (**A**) After 8 days of culture in collagen I Kif3a-i cells (−Tet) form spherical structures. Kif3a depleted cells (Kif3a-i +Tet) form clusters but no lumina. Scale Bars: 50 µm (**B**) Quantification of spherical structures in controls (pLVTH, n = 4), Kif3a-i1 (n = 3), and Kif3a-i2 (n = 3) cells in n independent experiments at day 7 (50 cellular structures per n). − and +Tet: controls: 47±6% vs. 49±3%; Kif3a-i1: 55±1% vs. 3±2%; Kif3a-i2: 43±2% vs. 25±5%; structures with one lumen. (**C**) Kif3a-i cells expressing the apical protein kidney-injury-molecule 1 (Kim-1) fused to CFP were grown in collagen I, fixed and stained for Hoechst (blue) and E-Cadherin (magenta). Non-induced Kif3a-i cells (−Tet) express Kim1 at the luminal side of spherical structures at day 7. Kif3a depleted cells (+Tet) show diffuse intracellular localization of Kim1. Scale Bars: 10 µm (**D**) Representative images show the effects of HGF treatment (10 ng/ml, 24 h) on Kif3a-i cells in the presence (+Tet) and absence (−Tet) of tetracycline. Kif3a depleted cells(+Tet) fail to form tubular extensions (lower right). (**E**) Quantification of extension formation in Kif3a-i and control cells (pLVTH) treated with tetracycline (n = 6, 12 fields of view for controls). Kif3a deficient cells (+Tet) form almost no extensions (n = 9, 18 fields of view, P<0.01).

The lack of Kif3a results in severe perturbations of embryonic morphogenesis [4]. We were interested if the MT defects we observed in migrating cells and its effects at the lateral membrane would be relevant for morphogenesis. We tested this in an vitro model of lumen formation that recapitulates many events analogous to mesenchymal to epithelial transition [23]. MDCK cells embedded in type I collagen develop spherical structures, where the apical membrane forms a central lumen and the basal membranes are oriented towards the surrounding matrix. Lumen formation is preceded by a complex series of polarizing events including the concentration of Par3 and aPKC at sites of ensuing lumen formation [24]. Strikingly, Kif3a-deficient MDCK cells grown in a collagen gel formed circular cellular aggregates but were unable to generate a central lumen, as opposed to the same cells in the absence of tetracycline or tetracycline-treated control cells (pLVTH) (Figure 6A and B). Also a second shRNA against Kif3a (Kif3a-i2) inhibited lumen formation, excluding off-target effects of Kif3a-i1. While expression of the apical protein Kim1 [16], [25] was restricted to the luminal side of the spheres in control cells, Kif3a depletion resulted in diffusely scattered expression of Kim1 throughout the cytoplasm (Figure 6C). MDCK cells in collagen grow tubular structures when exposed to HGF in an Eb1 dependent process [13], [26]. Since Eb1 tracking was slower and MT dynamicity was reduced in Kif3a deficient cells, we tested if Kif3a depleted cells were able to form tubular structures in response to HGF [23] but found virtually no tubular extensions (Figure 6D, E). These findings indicate that Kif3a is required for the formation of luminated three-dimensional structures.

### Lumenogenesis precedes cilia formation

**Figure 7:**
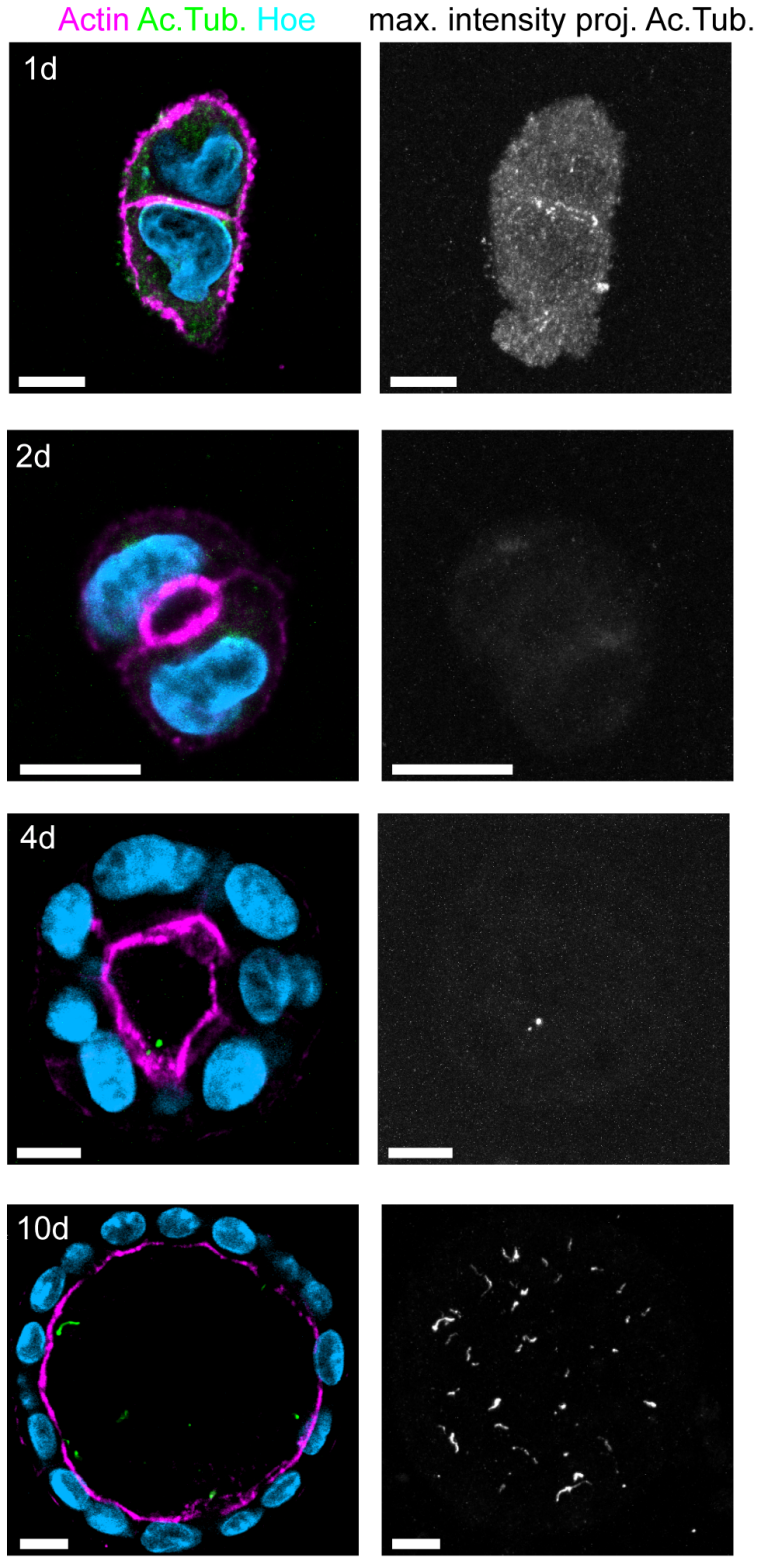
Lumen formation in collagen I precedes ciliogenesis. Wild type MDCK cells were grown in collagen I and stained for Hoechst, Actin and acetylated Tubulin at indicated time points. Merged section from one z-plane (left), projection of acetylated Tubulin from entire z-stack (right). At 24–48 h lumina are forming but no cilia are present. Cilia start appearing at day 4. At 10 days fully polarized lumina and cilia are present. Scale Bars: 10 µm.

Little is known about the role of MTs in 3D lumen formation. It is conceivable that cilia are required in this process and that the inability of Kif3a depleted cells to form lumina is due to their lack of cilia. Thus we investigated the temporal relationship between cilia formation and lumenogenesis. Primary cilia are present during G0 and get resorbed after entry in the cell cycle [27]. In two-dimensional culture, MDCK cells are unciliated at the time of seeding and form cilia beginning at day 4 [14]. We were interested if cilia preceded lumen formation of MDCK cells in collagen and stained MDCK cells in early stages of cyst formation with the cilia marker acetylated tubulin. This revealed that cilia were absent 2 days after seeding, at a time when the apical membrane was forming (Figure 7). Similar to cells in 2D culture the first cilia appeared on day 4, when fully formed cysts were present. These observations support the notion that the role of Kif3a in lumen formation is independent of cilia.

## Discussion and Conclusions

Our observations demonstrate that Kif3a has unexpected functions in regulating MT behaviour that are important for migration, lateral membrane specification and lumen formation. Two MT functions were most prominently regulated by Kif3a: MT dynamics and MT orientation. The leading edges of Kif3a depleted cells exhibited strikingly deviated MTs. Eb1-YFP live tracking of growing MTs in migrating control cells demonstrated that MTs grow directly into the leading edge. In Kif3a depleted cells, however, lamellipodia appeared smaller and MTs grew at highly oblique angles, avoiding the cell protrusions. Our finding of disordered MT polarity in Kif3a depleted cells is in agreement with a recently published study that found that Kinesin-2 is required for MT orientation in dendrites of drosophila neurons [28]. In this study Kinesin-2 acted in a complex with Eb-1 and APC to guide MTs at dendrite branches towards the cell body, and to reverse MT polarity in dendrites after injury to the axon, thus demonstrating that Kif3a is required in vivo for the polarization of MTs. Our findings together with this evidence argue for a general role of Kinesin-2 in MT orientation.

Kif3a interacts with the Par3/Par6/aPKC complex to regulate ciliogenesis [29]. The Par complex interacts with von-Hippel-Lindau protein (pVHL) which is a regulator of MT stability and has been shown to orient growing microtubules [30], [31]. The interaction of pVHL itself with MTs is orchestrated through binding to hetero-trimeric Kinesin-2 [32]. Therefore, in a hypothetical model Kif3a would recruit pVHL to stabilize, and the Par3/Par6/aPKC complex to orient microtubules. However, loss of pVHL leads to decreased pausing and increased dynamicity of MT [31], which is contrary to our observation of MT stunning in the absence of Kif3a. Another group of proteins recently implicated in MT orientation are the septin GTPases [33]. Future studies will need to address if Kif3a has a role in septin function.

MT dynamics in Kif3a depleted cells were characterized by a ‘stunned’ phenotype of decreased growth and shrinkage and increased pausing. It is unlikely that the profound alterations of MT behaviour after depletion of Kif3a can be explained by deficient transport of any single cargo molecule. However, one potential candidate is the +TIP protein APC. APC localization at the tips of membrane protrusions requires intact heterotrimeric kinesin 2 [34] and APC staining was absent from MT tips in our Kif3a depleted cells. Could the MT phenotype be explained by the failure of Kif3a deficient cells to target APC to MT tips? Similar to our findings, non APC-decorated MTs are characterized by a decreased growth rate and by increased pausing [35]. In addition, migration is impaired in APC depleted cells [36]. On the other hand non-APC decorated MTs display increased shortening [35], which deviates from our finding of decreased shortening in Kif3a depleted cells. Therefore, it is not clear at present which Kif3a dependent cargo is responsible for the MT defects we observed, and future studies will need to investigate different proteins transported by hetero-trimeric kinesin-2 to the leading edge and examine their individual effects on MT behaviour.

The function of Kif3a is not limited to migration. We found that Kif3a and microtubular dynamicity seem to have an important function in the generation of higher orders of polarity such as spheres and tubules in a collagen matrix. Kif3a depleted cells organized in normal appearing monolayers, with a regular distribution of junctional markers such as Zo-1, Par3, E-cadherin, β-catenin, Scrib, and apical Kim1. However, re-establishment of Par3 at the tight junction was delayed after break-down of the lateral membrane by temporarily withdrawing calcium from the medium, suggesting that Kif3a plays a role in tight junction assembly without being fully required in the setting of a plastic dish. On the other hand, Kif3a was indeed required for the formation of hollow spheres in collagen: Kif3a depleted cells predominantly formed cellular aggregates without lumina. Little is known about the role of microtubules in morphogenesis due to the difficulties of studying MT dynamics in three-dimensional structures. Live cell tracking of Eb1 in MDCK cells has demonstrated dynamic reorganization of the MT network during HGF induced epithelial remodelling of cysts in matrigel [13]. Moreover, Eb1 was required for HGF induced extensions from cysts into the surrounding matrix, but only modestly so for cyst formation itself. We observed a strong phenotype with almost no luminated structures of Kif3a depleted cells in collagen. It is therefore likely that Kif3a and MT dynamics are intimately involved in the complex interplay of redistribution of polarity molecules and vesicle trafficking during lumenogenesis [24]. These findings add to emerging role of MT dynamicity in epithelial morphogenesis.

Deletion of Kif3a results in the total absence of cilia and has been an important tool to study cilia function. Almost universally, phenotypic changes after deletion of Kif3a are interpreted as a consequence of missing cilia [4], [5], [37]–[41]. Constitutive deletion of essential cilia genes results in mid-embryonic lethality with severely disordered mesoderm development [4], [42]. Mutant Kif3a embryos are caudally truncated and display neural tube closure defects, a lack of limb primordia and pericardial edema [4]. The defects are attributed to disordered hedgehog (HH) signalling [5], however the phenotype is variable in different cell populations. This has been interpreted as a consequence of the fact that cilia have both activating and repressor roles in HH signalling [6]. We demonstrate that lack of Kif3a causes severe defects in an in-vitro model of morphogenesis. Interestingly, we found that early lumen formation in a three-dimensional matrix precedes cilia formation by approximately 2 days. This demonstrates that the lack of cilia is not the cause for the inability of Kif3a deficient cells to form a lumen and suggests that the lack of extra-ciliary Kif3a function is responsible for this finding. We therefore propose that the function of Kif3a during embryogenesis may be partially independent of cilia and extra-ciliary functions should be considered when interpreting phenotypes in Kif3a mutant animals.

Previous studies describe kinesin function on MT dynamic instability most prominently at the kinetocore, where kinesins, particularly Kinesin 8 and 13, guide microtubular disassembly, (reviewed in [43]). In non dividing cells, a MT plus-end stabilizing role has been described for Kif17 which constitutes homo-dimeric Kinesin-2 [9]. Kif17 stabilizes MT in conjunction with Eb1 and through recruitment of APC to MT tips. Functional heterotrimeric kinesin 2 is required for APC localization at neurite tips [34] and APC staining was absent from MT tips in our Kif3a depleted cells, providing indirect evidence that Kif17 and hetero-trimeric kinesin 2 may co-operate in the targeting of APC to its site of action. Kif3a and Kif17 have been described to have varying roles in primary cilia. In *C.elegans* neurons proximal transport is Kif3-mediated and transport at the distal segment occurs through Kif17 [44]. In zebrafish Kif17 has tissue specific roles, being required for photoreceptor formation but not for ciliogenesis in the pronephros [45]. The observations presented here raise the possibility that Kif3a and Kif17 may co-operate in MT transport outside cilia.

Our work demonstrates that Kif3a has a profound role in MT dynamics and organization that is important for cell migration and the formation of three-dimensional lumen carrying structures. These findings highlight the importance of further studies to detail the exact function of MTs in morphogenesis.

## Supporting Information

Figure S1 **(A)** Polyclonal MDCK cells generated by lentiviral gene transfer to express tetracycline inducible shRNA against Kif3a and a GFP reporter are subjected to western blot analysis 24 h and 48 h hours after seeding. Tetracycline treatment effectively suppresses the expression of Kif3a and causes co-expression of GFP. Loading is controlled by staining for γ-Tubulin. **(B)** Western Blot of lysates from MDCK cells with a second inducible shRNA against Kif3a (Kif3a-i2) demonstrates depletion of Kif3a upon tetracycline treatment. **(C)** Western Blot of MDCK cells with overexpression of flag-tagged human Kif3a (mutated shRNA target sequence) on MDCK.Kif3a-i cells. Upon tetracycline induction, Kif3a expression increases along with GFP, the latter indicating transcription of Kif3a-shRNA. **(D)** Western Blot of lysates from MDCK.Kif3a-i cells upon tetracycline treatment demonstrate downregulation of Kap, but same levels of acetylated-and detyrosinated tubulin. **(E)** Migrating MDCK cells were stained for APC (green), α-tubulin (magenta), and nuclei (blue). Punctuate staining of APC is present at plus-ends of MTs in Kif3a-i cells without tetracycline treatment (-Tet), but not in Kif3a-i depleted cells (+Tet). Scale Bars: 10 µm.
(TIF)

Figure S2 **(A)** Staining with antibodies against Zo-1, E-Cadherin, β-Catenin, Par3 and Scrib (white) reveals similar patterns in Kif3a-i cells grown on solid supports, both in the absence (-Tet) and presence (+Tet) of tetracycline. GFP appears in the merged image when Kif3a-i cells express the shRNA construct. Central Par3 signal represents staining at the mother centriole, as it has been described In the Par3 and Scrib stained panels GFP fluorescence is absent due to fixation with methanol. Scale bars: 20 µm. Lower images: Kif3a-i cells were stably transduced with the apical protein Kim1-CFP. Confocal z-stacks and xz-sectioning reveal localization of Kim1-CFP at the apical membrane. Scale Bars: 10 µm.
(TIF)

Table S1 **Quantification of microtubule behaviour in MDCK.Kif3a-i cells without or with tetracycline.**
(XLSX)

Video S1 **Sheet Migration in Kif3a-deficient cells (** **Figure 1** **).** Kif3a-i cells grown to confluence for two days without tetracycline (-Tet, left) close the wound after injury of the monolayer. Kif3a deficient cells (+Tet, right) have a severe migration defect (phase contrast images).
(MOV)

Video S2 **Kif3a-YFP associates with microtubule plus-ends during migration in MDCK cells.** MDCK cells were stably transduced with Kif3a-YFP and α-Tubulin-CFP, grown to confluence for two days, injured and migrating cells (six hours after wounding) imaged by dual camera TIRF microscopy. Kif3a-YFP signals are seen along MTs and at plus ends of MTs. Images were collected every two seconds for three minutes.
(MOV)

Video S3 **Eb1-YFP in migrating MDCK.Kif3a-i cells.** MDCK.Kif3a-i cells expressing Eb1-YFP cells were incubated with or without Tet and Eb1 dynamics were assessed in migrating cells six hours after wounding. In the absence of Tet Eb1-tracks lead perpendicularly into the leading edge, but they move parallel to the wound margin in Kif3a depleted cells.
(MOV)

Video S4 **Microtubule dynamics in migrating MDCK.Kif3a-i cells.** MDCK.Kif3a-i cells expressing α-Tubulin-YFP cells were incubated with or without Tet and microtubules were observed in migrating cells six hours after wounding to quantify MT dynamics. The MT ends appear ‘stunned’ in Kif3a-deficient cells.
(MOV)
