# Correction: Kif3a Guides Microtubular Dynamics, Migration and Lumen Formation of MDCK Cells

**DOI:** 10.1371/annotation/1eee5a30-6970-4b13-8369-a3158b79a426

**Published:** 2013-09-09

**Authors:** Christopher Boehlke, Fruzsina Kotsis, Bjoern Buchholz, Christian Powelske, Kai-Uwe Eckardt, Gerd Walz, Roland Nitschke, E. Wolfgang Kuehn

Two funding organizations were incorrectly omitted from the Funding Statement. The following sentence should be included with the Funding Statement: "The article processing charge was funded by the German Research Foundation (DFG) and the Albert-Ludwigs-University Freiburg through the funding programme 'Open Access Publishing'." 

