# Supplementary figures and images for: Kif3a Guides Microtubular Dynamics, Migration and Lumen Formation of MDCK Cells
Source: PLoS One. 2013 May 1;8(5):e62165. doi: 10.1371/journal.pone.0062165 (PMC3641035; doi:10.1371/journal.pone.0062165)

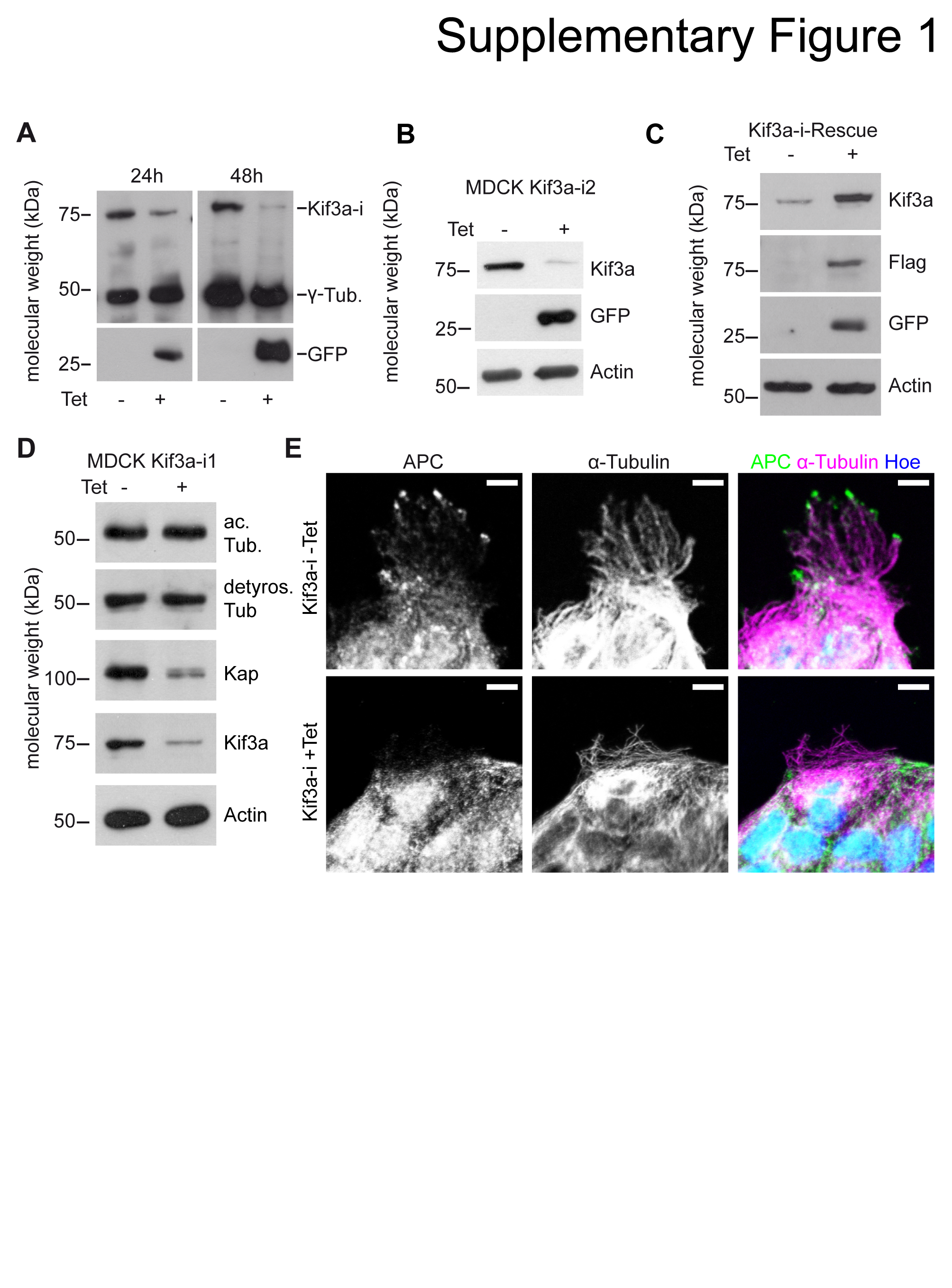

Supplement: Figure S1 — (A) Polyclonal MDCK cells generated by lentiviral gene transfer to express tetracycline inducible shRNA against Kif3a and a GFP reporter are subjected to western blot analysis 24 h and 48 h hours after seeding. Tetracycline treatment effectively suppresses the expression of Kif3a and causes co-expression of GFP. Loading is controlled by staining for γ-Tubulin. (B) Western Blot of lysates from MDCK cells with a second inducible shRNA against Kif3a (Kif3a-i2) demonstrates depletion of Kif3a upon tetracycline treatment. (C) Western Blot of MDCK cells with overexpression of flag-tagged human Kif3a (mutated shRNA target sequence) on MDCK.Kif3a-i cells. Upon tetracycline induction, Kif3a expression increases along with GFP, the latter indicating transcription of Kif3a-shRNA. (D) Western Blot of lysates from MDCK.Kif3a-i cells upon tetracycline treatment demonstrate downregulation of Kap, but same levels of acetylated-and detyrosinated tubulin. (E) Migrating MDCK cells were stained for APC (green), α-tubulin (magenta), and nuclei (blue). Punctuate staining of APC is present at plus-ends of MTs in Kif3a-i cells without tetracycline treatment (-Tet), but not in Kif3a-i depleted cells (+Tet). Scale Bars: 10 µm. (TIF) [file pone.0062165.s001.tif]

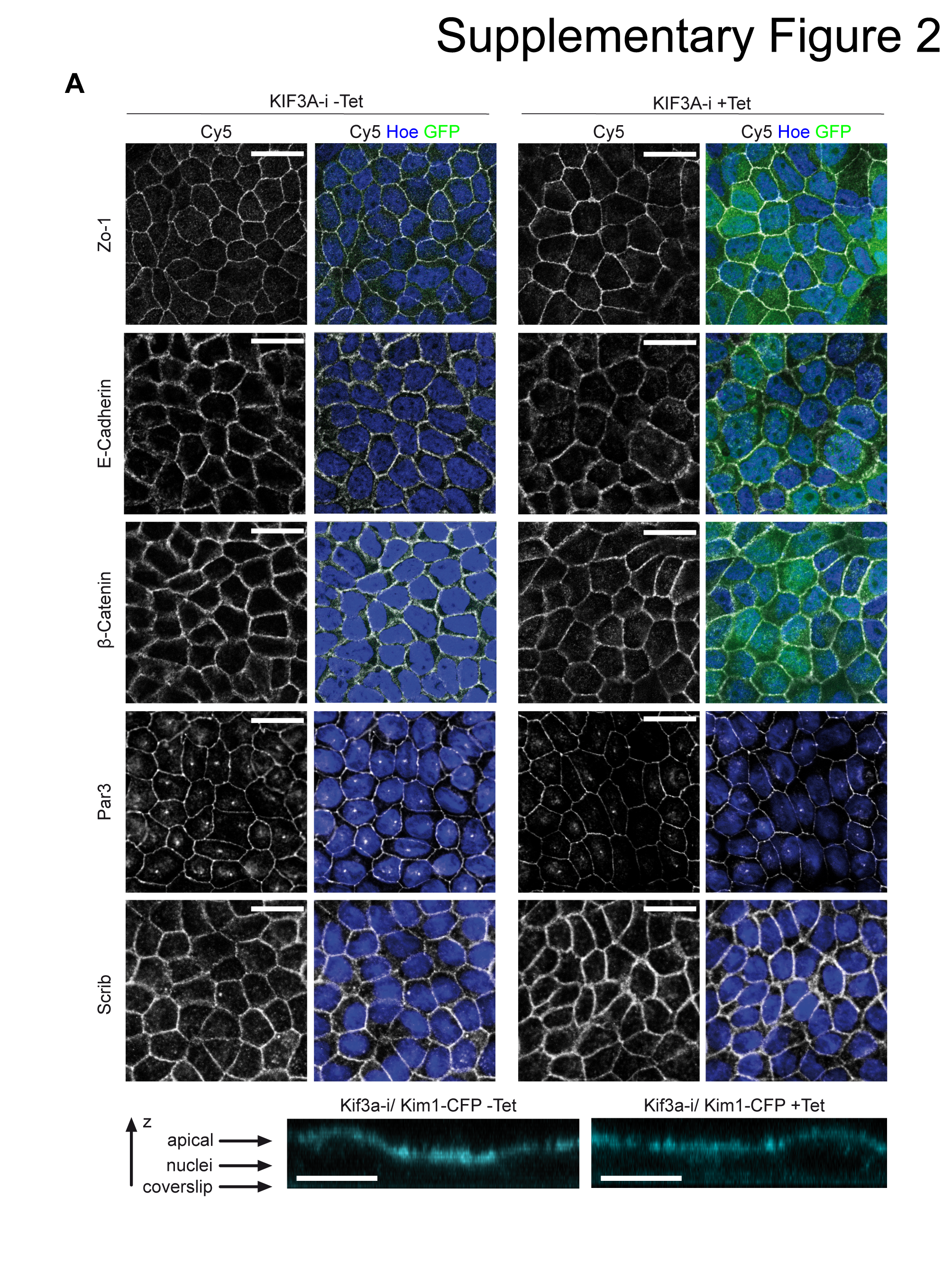

Supplement: Figure S2 — (A) Staining with antibodies against Zo-1, E-Cadherin, β-Catenin, Par3 and Scrib (white) reveals similar patterns in Kif3a-i cells grown on solid supports, both in the absence (-Tet) and presence (+Tet) of tetracycline. GFP appears in the merged image when Kif3a-i cells express the shRNA construct. Central Par3 signal represents staining at the mother centriole, as it has been described In the Par3 and Scrib stained panels GFP fluorescence is absent due to fixation with methanol. Scale bars: 20 µm. Lower images: Kif3a-i cells were stably transduced with the apical protein Kim1-CFP. Confocal z-stacks and xz-sectioning reveal localization of Kim1-CFP at the apical membrane. Scale Bars: 10 µm. (TIF) [file pone.0062165.s002.tif]
